# Ventral Tegmental Area Amylin Receptor Activation Differentially Modulates Mesolimbic Dopamine Signaling in Response to Fat versus Sugar

**DOI:** 10.1523/ENEURO.0133-24.2024

**Published:** 2024-06-07

**Authors:** Rohan V. Bhimani, Lily Rzepecki, Jinwoo Park, Elizabeth G. Mietlicki-Baase

**Affiliations:** ^1^Neuroscience Program, University at Buffalo, State University of New York, Buffalo, New York 14214-3005; ^2^Department of Biotechnical and Clinical Laboratory Sciences, University at Buffalo, State University of New York, Buffalo, New York 14214-3005; ^3^Department of Pharmacology and Toxicology, University at Buffalo, State University of New York, Buffalo, New York 14214-3005; ^4^Department of Exercise and Nutrition Sciences, University at Buffalo, State University of New York, Buffalo, New York 14214-3005; ^5^Center for Ingestive Behavior Research, University at Buffalo, State University of New York, Buffalo, New York 14214-3005

**Keywords:** macronutrient, nucleus accumbens, palatable food, reward, voltammetry, VTA

## Abstract

Amylin, a pancreatic hormone that is cosecreted with insulin, has been highlighted as a potential treatment target for obesity. Amylin receptors are distributed widely throughout the brain and are coexpressed on mesolimbic dopamine neurons. Activation of amylin receptors is known to reduce food intake, but the neurochemical mechanisms behind this remain to be elucidated. Amylin receptor activation in the ventral tegmental area (VTA), a key dopaminergic nucleus in the mesolimbic reward system, has a potent ability to suppress intake of palatable fat and sugar solutions. Although previous work has demonstrated that VTA amylin receptor activation can dampen mesolimbic dopamine signaling elicited by random delivery of sucrose, whether this is also the case for fat remains unknown. Herein we tested the hypothesis that amylin receptor activation in the VTA of male rats would attenuate dopamine signaling in the nucleus accumbens core in response to random intraoral delivery of either fat or sugar solutions. Results show that fat solution produces a greater potentiation of accumbens dopamine than an isocaloric sucrose solution. Moreover, activation of VTA amylin receptors elicits a more robust suppression of accumbens dopamine signaling in response to fat solution than to sucrose. Taken together these results shed new light on the amylin system as a therapeutic target for obesity and emphasize the reinforcing nature of high-fat/high-sugar diets.

## Significance Statement

Mesolimbic amylin receptor activation has strong anorectic effects, with a more robust suppression of fat versus sucrose intake. However, the neurochemical mechanisms underlying these differential effects remain unclear. The results of these studies demonstrate that pharmacological activation of amylin receptors in the ventral tegmental area attenuates dopamine signaling in the nucleus accumbens core evoked by intraoral administration of isocaloric fat or sucrose solution, but with a greater suppression of the fat-enhanced dopamine. These findings provide a potential physiological basis for the differential amylin-mediated intake responses toward these nutrients and further highlight this system as a promising therapeutic target for obesity.

## Introduction

Obesity remains a major global public health issue, despite advances in approved pharmacotherapies to treat this disease. In trying to identify novel physiological targets for treating obesity, the amylin system stands out as a promising candidate. Produced by the pancreas and cosecreted with insulin, amylin acts to suppress feeding and promote satiation [reviewed by Boyle et al. (2022)], and long-acting amylin agonists are currently under investigation as potential candidates for obesity treatment due to their ability to reduce body weight (Chakhtoura et al., 2023).

Amylin receptors consist of a calcitonin receptor (CTR) in association with a receptor activity modifying protein (RAMP; Pioszak and Hay, 2020). CTRs and RAMPs are expressed widely throughout the central nervous system, and amylin binding has been observed in many sites in the brain (Christopoulos et al., 1995; Paxinos et al., 2004), suggesting that amylin may act on a distributed network of central receptors to promote negative energy balance and exert other physiological and behavioral effects. A substantial body of literature has demonstrated the key role of area postrema amylin receptors in mediating the energy balance effects of amylin (Lutz et al., 1998, 2001; Mollet et al., 2004), but mesolimbic sites have also come into focus as relevant to amylin-mediated hypophagia. In the mesolimbic system, the ventral tegmental area (VTA) stands out as a pharmacologically and physiologically relevant site of action for amylin, and activation of amylin receptors in the VTA potently suppresses food intake, especially the intake of palatable foods (Mietlicki-Baase et al., 2013, 2015). This effect appears to be mediated at least in part by changes in dopamine (DA) signaling. Indeed, prior findings demonstrate that direct VTA administration of the amylin receptor/CTR agonist salmon calcitonin (sCT) in rats reduces the NAc core (NAcC) phasic DA response to random delivery of a sucrose pellet, and pharmacological activation of NAcC DA receptors attenuates the intake-suppressive effect of VTA sCT (Mietlicki-Baase et al., 2015), highlighting the importance of VTA→NAcC dopaminergic projections in the ability of VTA amylin receptor activation to suppress feeding.

Much of the published data on feeding effects of VTA amylin receptor signaling focus on intake of high-carbohydrate food (i.e., sucrose) or mixed-macronutrient diets like chow or a high-fat/high-carbohydrate diet, but there are some findings indicating that mesolimbic amylin receptor activation may more robustly impact the intake of palatable fat versus carbohydrate solutions. In a series of studies comparing intake of different concentrations of sucrose and Intralipid, including isocaloric solutions each providing 1 kcal/ml, more durable or potent suppression of fat intake compared with sucrose intake was observed after intra-VTA injection of sCT (Mietlicki-Baase et al., 2017). However, the mechanism underlying this effect is unclear.

Based on the evidence demonstrating the relevance of mesolimbic dopaminergic signaling in the NAcC for the intake-suppressive effects of VTA amylin receptor activation, and the robust suppression of fat intake by intra-VTA sCT injection, we hypothesized that DA signaling in the NAcC in response to intraoral administration of a fat solution would be more strongly suppressed by VTA sCT than DA release to delivery of sucrose. We tested this in awake, behaving male rats using fast-scan cyclic voltammetry (FSCV) coupled with carbon-fiber microelectrodes to assess rapid changes in phasic DA concentration in the NAcC. Our findings show that intraoral delivery of isocaloric fat and sucrose solutions both enhance NAcC DA transmission; however, fat solutions potentiate DA levels in the NAcC to a greater degree. Additionally, VTA amylin receptor activation with sCT more robustly reduces the NAcC DA signal elicited by fat compared with sucrose.

## Materials and Methods

### Animals

Thirty-five male Sprague Dawley rats (280–350 g, aged ∼8 weeks at the time of arrival) were obtained from Charles River Laboratories. Rats were initially pair housed in a polycarbonate cage with a wire lid holding rat chow within a humidity- and temperature-controlled environment with a 12 h light/dark cycle (lights on at 07:00 A.M.). Food and water were available *ad libitum*. Water was delivered through an animal watering system via a small opening in each cage. All procedures related to handling and caring for animals were performed in accordance with the *Guide for the Care and Use of Laboratory Animals* (8th Edition, 2011, US National Research Council) and were approved by the Institutional Animal Care and Use Committee of the University at Buffalo (Protocol CLS15022Y).

### Drugs and reagents

The amylin receptor agonist salmon calcitonin (Bachem, #4033011) was dissolved in artificial cerebrospinal fluid (aCSF, Harvard Apparatus, #59-7316). Drug dose was selected based on previous studies showing that intra-VTA administration of 0.04 µg sCT suppresses *ad libitum* intake of fat and sucrose at the concentrations used in the present work (Mietlicki-Baase et al., 2017) and is subthreshold for prolonged effects on feeding when delivered directly to the cerebroventricle (Mietlicki-Baase et al., 2013). Importantly, VTA injection of this dose of sCT also does not cause long-term effects on locomotor activity (Mietlicki-Baase et al., 2013) nor does it produce anxiety-like behaviors (Mietlicki-Baase et al., 2017). In vitro calibration of carbon-fiber microelectrodes was performed in a Tris buffer solution, pH 7.4, containing the following (in mM): 15 Tris, 140 NaCl, 3.25 KCl, 1.2 CaCl_2_, 1.25 NaH_2_PO_4_, 1.2 MgCl_2_, and 2.0 Na_2_SO_4_ in double distilled water with different concentrations of dopamine (Sigma, #H8502; Bhimani et al., 2021).

### Intraoral catheter fabrication

Intraoral catheters were fabricated in-house and consisted of 6 cm pieces of polyethylene (PE)-100 tubing. Tubing was heated with a soldering iron and cooled on an anvil to create a disk shape. A pin was inserted inside the flat end of the PE tubing to open the hole. A 3 inch guide needle for catheter insertion was made by modifying an 18 gauge catheter needle (Terumo Medical Products, #SR*FF1851). Catheters and guide needles were cold sterilized for 24 h in glutaraldehyde prior to surgery. Washers to secure the tubing inside the oral cavity consisted of polytetrafluoroethylene sheets (1 mm thickness) with a hole punched in the center (1.8 mm for the oral cavity and 1.5 mm for the external washer).

### Surgical procedures

Rats were anesthetized with an intraperitoneal injection of ketamine (85 mg/kg) and xylazine (10 mg/kg). Intraoral and stereotaxic surgeries for electrochemical measurements were performed as described previously (J. Park et al., 2012). Fur on the skull and oral catheter exit site was depilated and cleaned with chlorhexidine and isopropyl alcohol prior to surgery. A washer was placed on the oral catheter. The guide needle was next introduced into the catheter, and then the catheter was inserted lateral to the first maxillary molar with the washer secured against the molar. The guide needle exited posterior and lateral to the ears and was then removed and replaced with the small diameter washer and secured in place with surgical glue (Vetbond, 3 M). Catheters were flushed with sterile saline to verify patency.

Rats were transferred to and placed in a stereotaxic frame (David Kopf Instruments) and the scalp was swabbed with iodine and ethanol. Preincision local anesthesia was induced by a subcutaneous injection of ropivacaine (1.6 mg/kg), and a central incision was made to expose the skull surface. Anteroposterior (AP), mediolateral (ML), and dorsoventral (DV) coordinates were obtained from the rat brain atlas (Paxinos and Watson, 2006) and referenced from bregma. One-millimeter holes were drilled in the skull for implantation of bone screws and electrodes/cannulas. A guide cannula (#MD-2251, Bioanalytical Systems) for loading a micromanipulator containing a fresh carbon-fiber microelectrode (neurochemical sensor) was implanted above the nucleus accumbens core (NAcC; AP: +1.3 mm, ML: 1.4 mm). A 26 gauge guide cannula (Plastics One, C315GRL) for drug delivery was implanted above the VTA (AP: −5.8 mm; ML: 0.8 mm) and mounted with a dummy cannula (Plastics One, C315G/SPC) until the day of recording. A Ag/AgCl reference electrode was implanted on the contralateral cortex. Immediately following surgery, rats received subcutaneous (SC) fluids (isotonic saline, 10 ml/kg) and carprofen (5.0 mg/kg). Animals were further administered carprofen (5.0 mg/kg, s.c., for 2 d) and allowed to recover for 1 week before electrochemical and behavioral recordings. Following surgery, all rats were housed individually in clean cages to prevent damage to the surgical site and fed mealed rat chow (Envigo, # T.2018CM.15) mixed with water to ensure rats did not drop below presurgery body weight. Oral catheters were also flushed daily with water.

### Experimental design

One week after recovery from surgery, rats were placed in a custom-built operant chamber designed for voltammetric recordings. At the start of the behavioral session, white noise was activated to control for potentially interfering ambient noise, and the house light was turned on to observe any behavioral changes. Naturally occurring phasic dopamine release in the NAcC was mapped by lowering the electrode in 0.2–0.3 mm intervals and its response to administering sucrose or Intralipid (Baxter Healthcare) to determine at what depths naturally occurring phasic dopamine release was evoked maximally, similar to our previous studies (Mietlicki-Baase et al., 2015; Bhimani et al., 2021). Because we did not use a stimulating electrode to map electrically evoked dopamine release sites and minimize tissue damage, rats received 1–2 trials of solution (either sucrose or Intralipid depending on experiment) at a maximally and naturally occurring phasic dopamine release site (no more than two sites) instead during mapping. This way of mapping allowed investigation of maximal phasic dopamine responses to sucrose or fat along the dorsal-ventral axis of the NAcC. Microelectrode position did not change following mapping. After a variable time interval (30–120 s), an infusion pump (Med-Associates, PHM-200) delivered ∼200 μl of solution [either 25% sucrose or 10% Intralipid, both 1 kcal/ml] for 3.5 s through the intraoral cannula. Rats received 25 trials of solution first and then 100 nl of vehicle (aCSF) or the amylin receptor agonist (sCT) was delivered to the VTA via intraparenchymal injection. Thirty minutes later, rats received an additional 25 trials. Rats remained connected to the FSCV system during the entire experiment, which additionally allowed for determining if sCT alone had any effects on phasic dopamine transmission.

### Voltammetric procedures

Glass-encased, cylindrical carbon-fiber microelectrodes with an exposed length of 75–100 μm T-650 carbon fiber (7 μm in diameter) and reference electrodes were prepared as described previously (Bhimani et al., 2021). FSCV was computer-controlled using TH-1 software (University of North Carolina Department of Chemistry Electronic Shop). A triangular scan (−0.4 to +1.3 V, 400 V/s) was low-pass filtered at 2 kHz and repeated every 100 ms. Data were digitized and stored on a computer with software written in LABVIEW (National Instruments). Background-subtracted cyclic voltammograms were obtained by digitally subtracting voltammograms collected during infusion of solution from those collected during baseline recording. Voltammetric responses were viewed as color plots with the abscissa as voltage and the ordinate as acquisition time and the current encoded in color (Bhimani et al., 2022). Because the carbon-fiber microelectrode was used to lesion the brain, thus marking the recording site, this precluded postcalibration of the sensitivity of the same electrode. Instead, we used postcalibration factors/carbon-fiber area (10.5 ± 0.4 pA/[μmol/L × μm^2^]) on the basis of the average response obtained from multiple electrodes of different sizes (between 75 and 100 μm length) that were exposed to rat brains to monitor NAc DA for at least 5 h (Pauly et al., 2023). Each calibration factor was determined with five DA concentration standards. The calibration factors are scaled to the electrode length that varied between 75 and ∼100 μm. Before the experiment, the length of the exposed carbon fiber was measured.

### Histology

**Figure 1.**
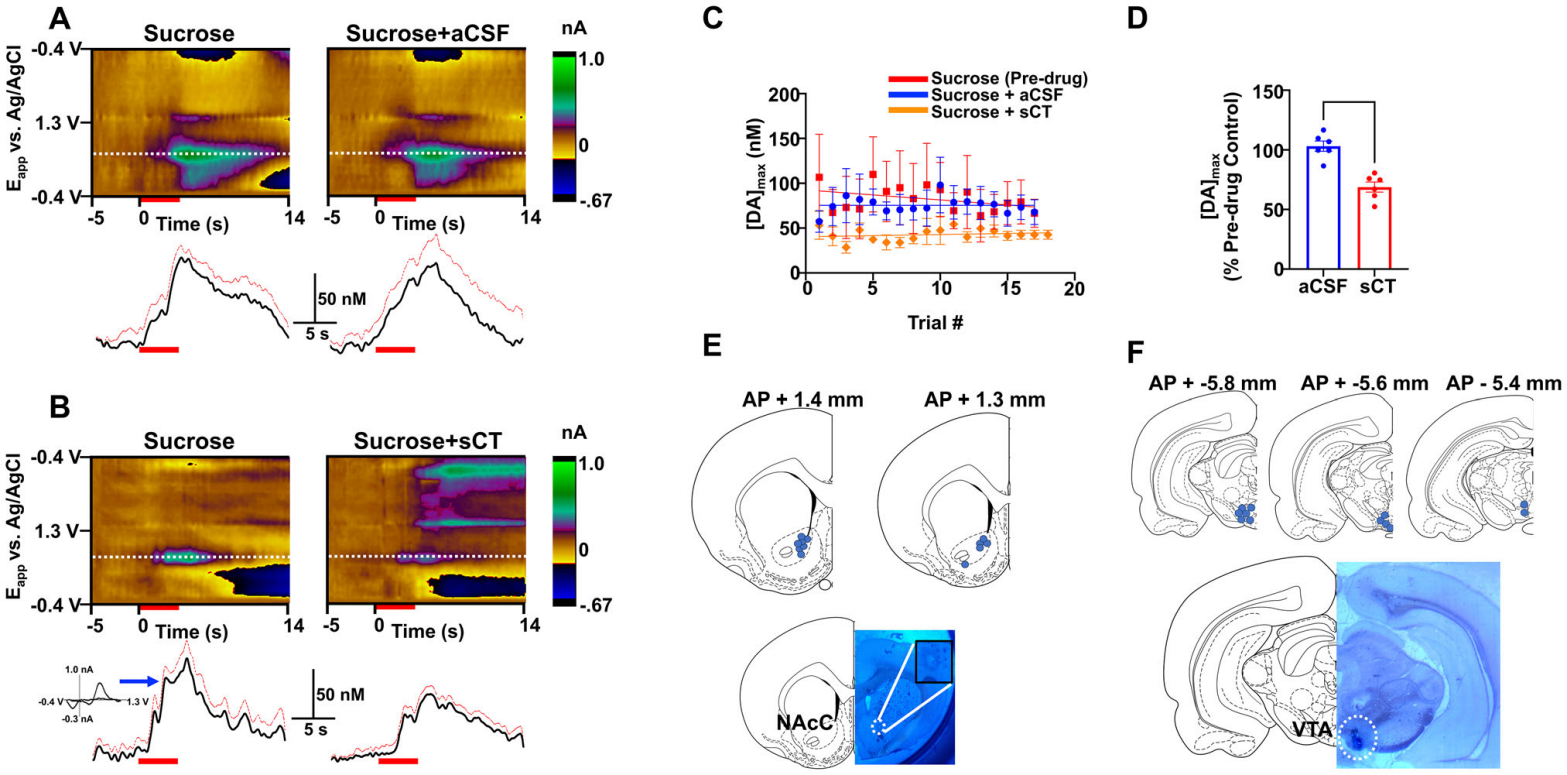
Effects of VTA amylin receptor activation on modulating sucrose-enhanced DA in the NAcC. Average color plots (top) show background subtracted cyclic voltammograms collected 5 s before and 14 s after administration of sucrose from a single rat. DA concentration changes were seen in the color plot at the potential (*E*_app_) for DA oxidation (∼0.65 V, dotted white line) in response to intraoral sucrose administration before and after aCSF (***A***) or sCT (***B***) administration following principal component analysis. A representative average cyclic voltammogram is highlighted in Figure 1*B* from a point in the concentration versus time trace (indicated by blue arrow). Red line and red bar on the DA concentration trace indicates standard error and duration of sucrose infusion (4 s), respectively. ***C***, Trial by trial maximal DA responses ([DA]_max_) before [termed “Sucrose (Pre-drug)”] and after vehicle (aCSF, blue line) or amylin receptor agonist (sCT, orange line) administration. ***D***, sCT significantly decreased average maximal DA concentration in response to sucrose compared with vehicle (aCSF) administration. ***E***, Electrode recording sites in the NAcC for animals used in sucrose experiments (top) and representative electrolytical lesion (bottom). ***F***, sCT and aCSF injection sites for animals used in sucrose experiments (top) and representative dye infusion in the VTA (bottom) indicating where drug was injected. ***p < 0.001.

**Figure 2.**
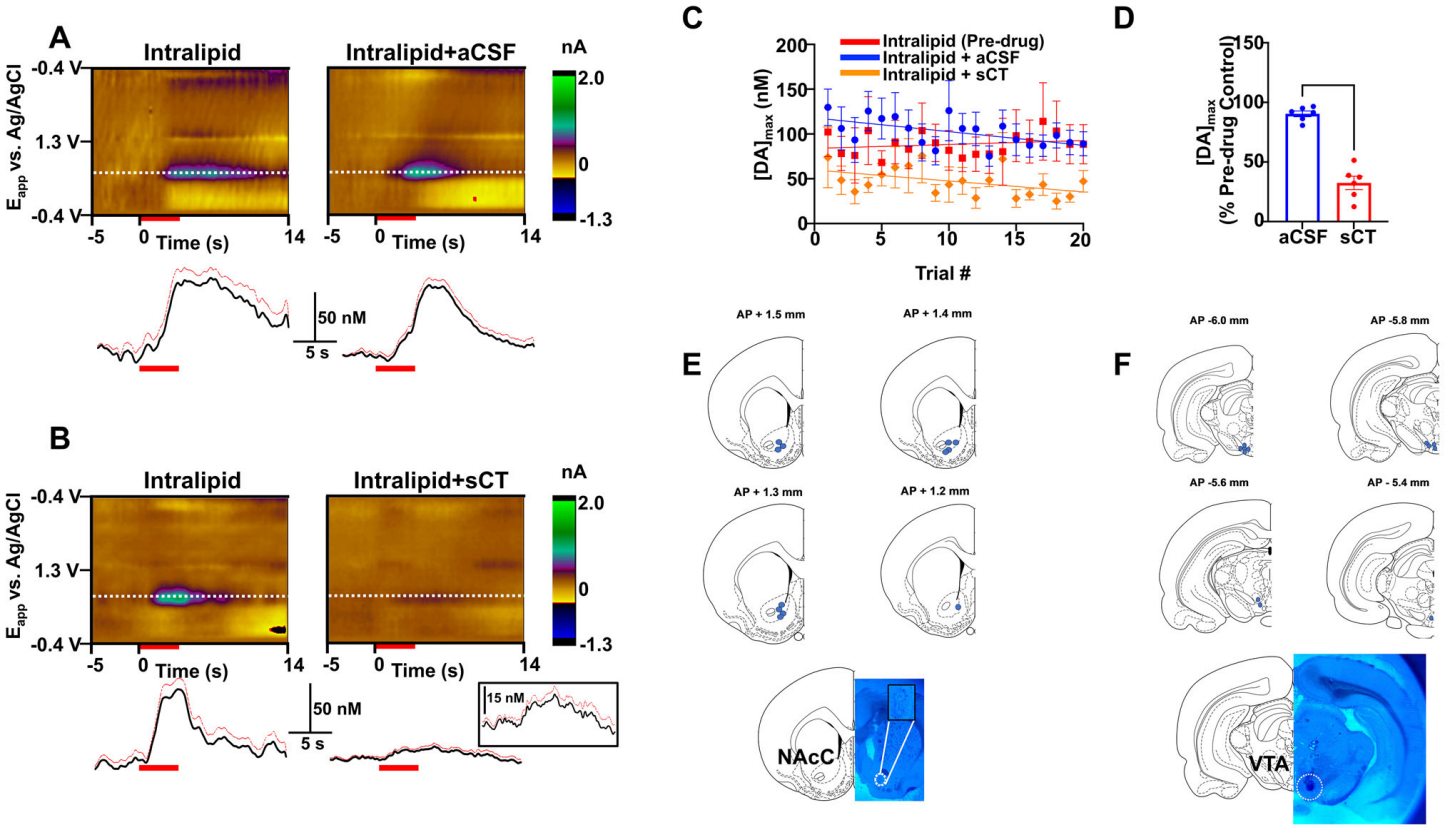
Effects of VTA amylin receptor activation on modulating Intralipid-enhanced DA in the NAcC. Average color plots (top) show background subtracted cyclic voltammograms collected 5 s before and 14 s after administration of Intralipid from a single rat. DA concentration changes were seen in the color plot at the potential (*E*_app_) for DA oxidation (∼0.65 V, dotted white line) in response to intraoral Intralipid administration before [termed “Intralipid (Pre-drug)”] and after aCSF (***A***) or sCT (***B***) administration following principal component analysis. The inset shows the concentration versus time trace following sCT administration at a higher magnification. Red line and red bar on the DA concentration trace indicates standard error and duration of Intralipid infusion (4 s), respectively. ***C***, Trial by trial maximal DA responses before and after vehicle (aCSF, blue line) or amylin receptor agonist (sCT, orange line) administration. ***D***, sCT significantly decreased average maximal DA concentration in response to sucrose compared with vehicle (aCSF) administration. ***E***, Electrode recording sites for animals used in sucrose experiments (top) and representative electrolytical lesion (bottom). ***F***, sCT and aCSF injection sites for animals used in sucrose experiments (top) and representative dye infusion in the VTA (bottom) indicating where drug was injected. ****p < 0.0001.

At the end of experiments, all rats were deeply anesthetized with sodium pentobarbital (487.5 mg/kg), and the recording sites were verified by electrolytic lesions by applying constant current (20 μA for 10 s) to the carbon-fiber microelectrodes. Drug infusion sites were verified by injecting dye (Chicago sky blue, Sigma-Aldrich, C8679). Rats were transcardially perfused with 500 ml of phosphate-buffered saline (PBS, 4°C) followed by 350 ml of 10% buffered formalin phosphate (4°C). Brains were extracted from the skull, postfixed for 12 h in 10% buffered formalin phosphate at 4°C, and then cryoprotected in 30% sucrose in 0.1 M PBS for 72 h. Brains were sectioned on a freezing microtome at 35 μm, and sections were visualized on a light microscope to verify electrode placements and infusion sites. Figures 1 and 2*E*,*F* show electrode recording sites, representative electrolytical lesion sites, and dye injection sites from all rats used in these studies.

### Data and statistical analyses

This study was not preregistered and data are accessible by contacting the authors. The changes of basal DA concentration by fat or sugar solution over time was identified by a locally written principal component regression algorithm as described earlier (Bucher et al., 2013). A residual analysis procedure was used to verify that the cyclic voltammograms of the trials being predicted were consistent with the analyte cyclic voltammograms used for calibration. Only DA concentrations with signal-to-noise >5 were considered as fat- or sugar-evoked DA release. Any trials containing uncharacteristic variance larger than 95% of the noise of the training set were excluded from analysis. All rats had some trials excluded due to a high residual (above the level where 95% of the noise) leading to <25 total trials in the analysis (J. Park et al., 2012, 2013). Significant changes over time were evaluated with average baseline (−5.0 to 0 s relative to infusion onset) and maximal evoked DA concentration ([DA]_max_) during and after infusion (0.1–14 s). Drug effects were determined by comparing average DA concentration after microinfusions to predrug controls and using a two-tailed Student's *t* test to calculate the level of significance. Simple linear regression and one-way ANOVA were used to compare DA responses across trials. The Grubbs’ test was used to determine whether there were any outliers. One rat was found to be an outlier and excluded from analysis of predrug evoked DA responses to solution. GraphPad software version 10 was used to perform all analyses. A *p* value <0.05 was regarded as statistically significant. Data are represented as mean ± SEM. A total of 11 rats that showed off target recording/infusion sites or had cannula failure were excluded from analysis.

## Results

VTA DA signaling has been implicated in encoding the reward value of appetitive stimuli such as sucrose (Norgren et al., 2006; Alcaro et al., 2007; J. Park et al., 2012), and VTA amylin receptor activation suppresses DA signaling in the NAcC in response to sucrose pellet delivery (Mietlicki-Baase et al., 2015). However, activation of VTA amylin receptors potently suppresses fat intake as well (Mietlicki-Baase et al., 2017). It is unknown whether NAcC DA elicited by exposure to fat is similarly reduced by VTA amylin receptor activation. Furthermore, it is unclear whether NAcC DA responses produced by oral stimulation with fat versus sucrose are equivalent. Therefore, in the following experiments, rats received noncontingent random intraoral delivery of fat or isocaloric sucrose solutions before and after activation of VTA amylin receptors. Any trials that failed residual analysis during the principal component regression were discarded (J. Park et al., 2012, 2013).

One week following combined intraoral/FSCV surgeries, rats were placed in the FSCV behavioral chambers. A micromanipulator containing a carbon-fiber microelectrode was positioned above the NAcC at −6.0 mm below the skull surface. The electrode was then lowered in 0.2–0.3 mm increments between 6.0 and 7.5 mm ventral until naturally occurring phasic DA release was observed and its response to delivery of a 25% sucrose solution (200 μl, 3.5 s). Once maximal phasic DA release and its response to sucrose in the NAcC was seen, rats received 25 trials of sucrose followed by intraparenchymal injection of either vehicle (aCSF) or the amylin receptor agonist (sCT) into the VTA. Thirty minutes later, rats were subjected to another 25 trials of sucrose. Any trials that failed residual analysis during the principal component regression were discarded (see Materials and Methods, Data and statistical analyses).

Figure *1*, *A* and *B*, shows representative average color plots (top) and DA concentration versus time traces (bottom) from a single animal following intraoral delivery of sucrose before and after infusion of aCSF and sCT into the VTA, respectively. An average cyclic voltammogram identifying the signal as catecholamine is shown in Figure 1*B* (blue arrow). It is noteworthy that the major catecholamine at the coordinate used in this study is dopamine (over 99%; Kilts and Anderson, 1986). Figure 1*C* shows average maximal DA concentrations ([DA]_max_) by trial following sucrose administration before and after delivery of aCSF or sCT from multiple rats. Responses to sucrose were consistent across trials. Intraparenchymal injection of sCT into the VTA caused a significant decrease in maximal DA response to sucrose (*t*_(10)_ = 5.076; *p *= 0.0002; Fig. 1*B*,*D*). Electrode and drug infusion sites are shown in Figure 1*E*,*F*.

Next, we investigated whether VTA sCT infusion also suppressed NAcC DA responses to oral delivery of fat. Figure 2, *A* and *B*, shows average color plots and DA concentration versus time traces from a single animal before and after administration of aCSF and sCT, respectively. VTA amylin receptor activation caused a significant decrease (∼68%) in DA release after Intralipid administration (*t*_(10)_ = 9.596; *p *< 0.0001; Fig. 2*B*,*D*). Maximal DA responses to Intralipid were reproducible across trials both before and after vehicle administration (Fig. 2*C*). Pharmacological activation of VTA amylin receptors caused a gradual but not statistically significant decrease in maximal DA concentration over time. Electrode and drug infusion sites are shown in Figure 2*E*,*F*.

**Figure 3.**
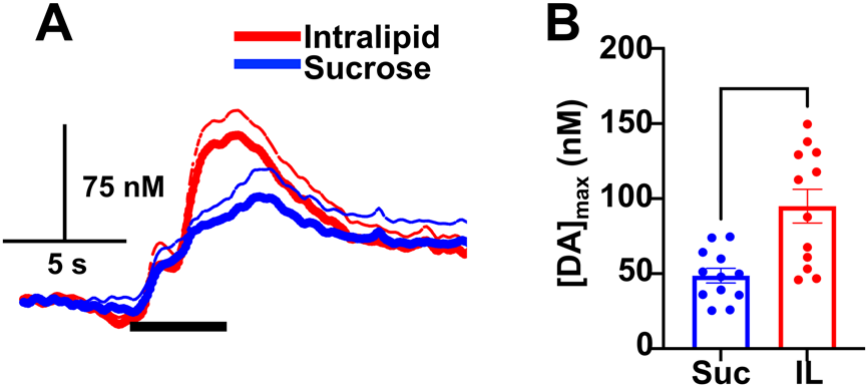
Comparison of sucrose (Suc) and Intralipid (IL) effects on NAcC DA. ***A***, Average DA concentration versus time traces across all animals for sucrose (blue) and Intralipid (red) administration. The dotted lines indicate standard error from mean (solid line). ***B***, Average maximal DA concentration (nM) changes in response to intraoral sucrose or Intralipid administration before sCT or vehicle (aCSF) injection. *p < 0.05.

Both sucrose and fat can enhance accumbens DA concentration, effects that have been demonstrated previously (Liang et al., 2006; Mietlicki-Baase et al., 2015) and that are recapitulated here. However, direct comparisons between how isocaloric sucrose and fat solutions enhance DA release are limited. Therefore, we determined how isocaloric sucrose and fat solutions impact DA signaling. Comparisons were made by analyzing average baseline responses from all rats prior to vehicle or drug infusion. Figure 3*A* shows average DA concentration versus time traces (before drug administration) for sucrose (*n* = 12 rats) and Intralipid (*n* = 12 rats) across all animals. Intralipid administration enhanced maximal DA release ([DA]_max_) significantly greater than sucrose (*t*_(22)_ = 3.790; *p *= 0.0005; Fig. 3*B*). Average maximal DA concentration changes for both sucrose and Intralipid after aCSF or sCT are shown in Table 1. In addition to the larger DA increase evoked by fat compared with sucrose, the NAcC DA release evoked by intraoral delivery of fat was significantly more blunted by sCT than the response evoked by IO sucrose delivery (*t*_(10)_ = 4.040; *p *= 0.0012; Table 1).

**Table 1.** Average maximal DA concentration changes ([DA]_max_, nM) following intraoral administration of sucrose or Intralipid after VTA injection of either aCSF or sCT

Drug	[DA]_max_ to sucrose (*n* = 6 rats/condition)	[DA]_max_ to intralipid (*n* = 6 rats/condition)
aCSF	59.7 ± 8.1 nM	88.8 ± 10.5 nM
sCT	33.8 ± 3.2 nM	15.8 ± 3.1 nM

Data are presented as means ± SEM.

**Figure 4.**
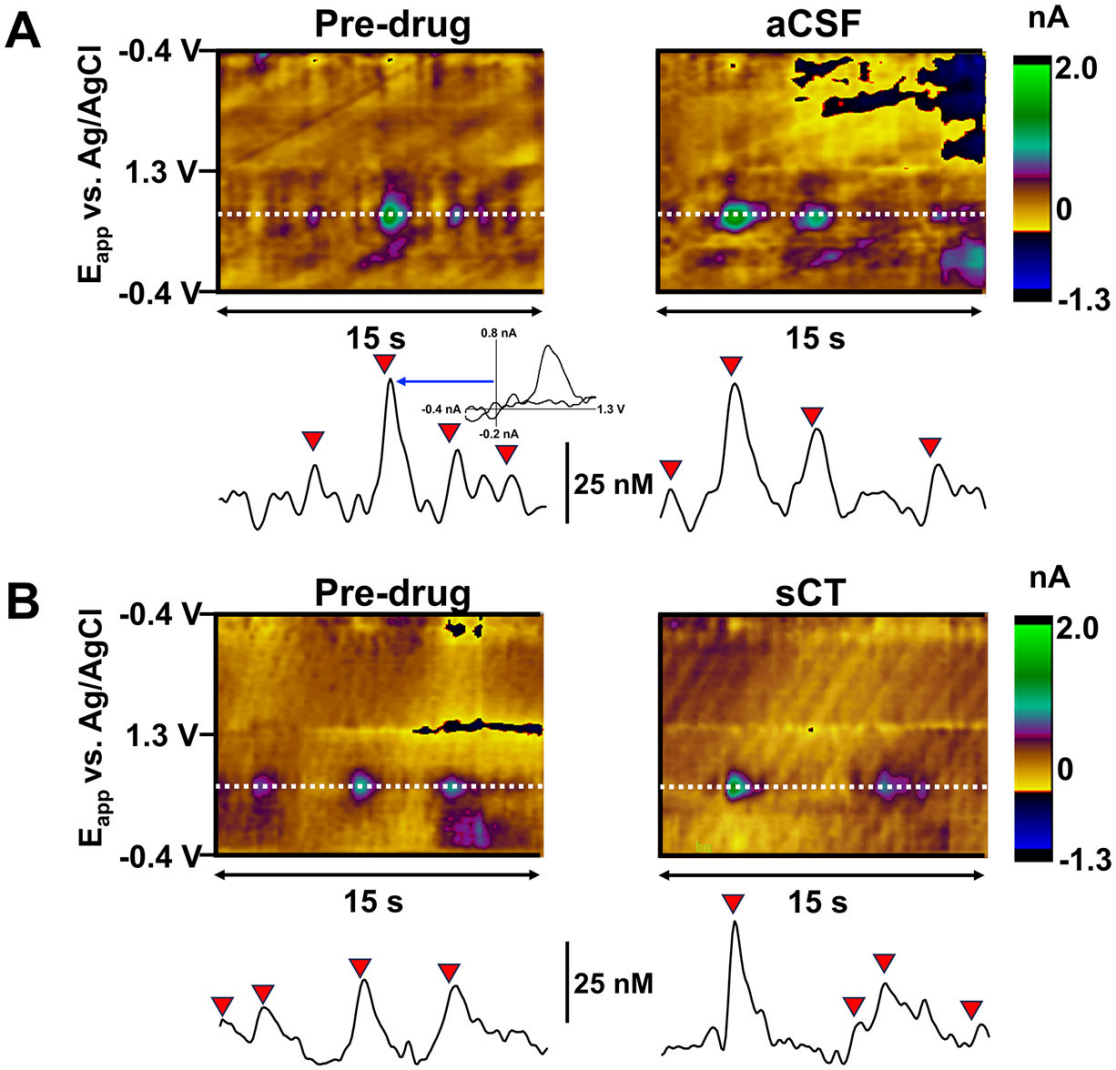
Effects of the amylin receptor agonist (sCT) on naturally occurring phasic dopamine transmission. *A***,** *B***,** Representative color plots show background subtracted cyclic voltammograms collected before (left) and 30 min after (right) administration of aCSF (top) or sCT (bottom) from single rats. DA concentration changes were seen in the color plot at the potential (*E*_app_) for DA oxidation (∼0.65 V, dotted white line). Red reversed triangles indicate phasic dopamine transients. A representative cyclic voltammogram from a phasic dopamine transient is highlighted in Figure 4*A* from a point in the concentration versus time trace (indicated by blue arrow).

While the effects of sCT on sucrose- and fat-evoked DA were very pronounced, we investigated whether sCT also produced any generalized decrease of naturally occurring phasic DA transmission. Figure 4 shows representative color plots and concentration versus time traces from a single rat before and after intra-VTA injection of aCSF (Fig. 4*A*) or sCT (Fig. 4*B*). Neither aCSF nor sCT significantly impacted the concentration or frequency of phasic DA transmission. Average DA transient concentrations and frequencies from all rats are depicted in Table 2. Importantly, our previous studies have also demonstrated that intra-VTA sCT does not produce prolonged effects on locomotor activity or anxiety-like behaviors (Mietlicki-Baase et al., 2013, 2017). Although we did not systematically evaluate off-target behavioral outcomes in the present experiments, no gross behavioral changes were noted after intra-VTA sCT.

**Table 2.** Average naturally occurring phasic dopamine transient concentration (nM) and frequency (Hz) before and after VTA injection of either aCSF or sCT

Drug	Dopamine concentration (nM)	Frequency (Hz)
Predrug	43.9 ± 2.5	0.38 ± 0.03
aCSF	45.4 ± 0.2.7	0.37 ± 0.03
sCT	43.8 ± 2.4	0.37 ± 0.02

Data are presented as means ± SEM.

## Discussion

These studies demonstrate that intra-VTA administration of the amylin receptor agonist, sCT, suppressed the increase in NAcC DA elicited by intraoral delivery of either a sucrose solution or an isocaloric fat solution. Previously published data showed that a reduction in DA signaling in the NAcC is important for the hypophagic effects of VTA amylin receptor activation (Mietlicki-Baase et al., 2015). The current findings suggest that the magnitude of suppression of NAcC DA by VTA sCT is greater for fat than for sucrose, providing novel insight into how amylin receptor signaling can modulate mesolimbic DA responses to different palatable solutions.

The reduction in NAcC DA signaling in response to intraoral delivery of sucrose following VTA amylin receptor activation is similar to the outcome of a previous study examining the impact of sCT on phasic NAcC DA to random delivery of a sucrose pellet (Mietlicki-Baase et al., 2015). Both the current study and the previous study with sucrose pellets demonstrated ∼25% reduction in the NAcC DA signal to the respective stimuli after VTA sCT. However, our study builds on the previous findings by comparing responses to sucrose versus those to an isocaloric fat solution. Importantly, as sCT has been shown to have anorectic effects, in this study oral exposure to each solution was not contingent upon the animal's behavior (Lutz et al., 2000). Therefore, the direct effects of VTA amylin receptor activation on DA signaling in response to orosensory stimulation from fat or sucrose was determined without confounding effects related to changes in food-seeking behavior. Importantly, no changes in natural phasic dopamine transmission nor basal behavior were observed, similar to our previous open-field studies following intra-VTA injection of sCT demonstrating no prolonged suppression of locomotor activity of induction of anxiety-like behavior (Mietlicki-Baase et al., 2013, 2017). Previous findings show that VTA amylin receptor activation can elicit a greater suppression of the intake of fat solution compared with sucrose solution under different experimental paradigms (Mietlicki-Baase et al., 2017), and the potent suppression of the fat-associated DA response after VTA sCT observed here may provide mechanistic insight for this effect.

Few studies have examined accumbens dopaminergic responses to fat as an orosensory stimulus. Using microdialysis, it was shown that sham intake of corn oil produced increases in DA in the NAc shell (Liang et al., 2006) on a longer timescale of minutes, as opposed to seconds in the current study. Oral lipolysis is likely important for the detection of fatty solutions in the oral cavity (Kawai and Fushiki, 2003). Interestingly, high-fat diets have been shown to elevate DA in the NAc shell to a greater extent than do low-fat diets, and systemic injections of Intralipid elevate baseline DA concentration more than does glucose injection (Rada et al., 2012). Although prior investigation of DA responses to fat have focused on effects in the NAc shell, we focused in the present studies on the NAcC because of the established role of DA signaling in this site in mediating the hypophagic effects of VTA amylin receptor activation (Mietlicki-Baase et al., 2015). Our results show that DA signaling in the NAcC was enhanced significantly more by fat than sugar solutions despite similar caloric content. Although this means that there are distinct predrug DA concentrations evoked by the fat and sucrose solutions used here that could influence the interpretation of the comparative ability of VTA sCT to suppress the responses, we further analyzed the data as absolute DA concentration changes in addition to a percentage of predrug control (relative DA concentration changes) to account for the differences in drug effects (J. W. Park et al., 2017). This supported the outcome that the average fat-evoked DA concentration following sCT administration is still significantly attenuated more than the average sucrose-evoked DA signal after sCT (Table 1) and demonstrates that, at least at these concentrations, sCT suppresses fat-induced DA release more than it does sucrose-induced DA.

A notable limitation of the current set of studies is that these experiments were performed only in male rats. Indeed, most published work on the effects of mesolimbic amylin receptor signaling for energy balance control has examined responses exclusively in male subjects (Mietlicki-Baase et al., 2013, 2015, 2017). The effects of amylin on feeding and weight control in females remains an active area of investigation (Roth et al., 2007; Asarian and Geary, 2013; Maske et al., 2020), and it will be important in future work to determine whether VTA amylin receptor activation in females produces similar effects on NAcC DA responses to nutrient stimuli. It will also be informative to evaluate the influence of the estrous cycle on the effects of amylin signaling, because estrogens can exert both within-cycle and chronic effects on the ability of amylin to suppress feeding (Trevaskis et al., 2010; Asarian and Geary, 2013).

Another important consideration of the present findings is that the effects of VTA sCT on NAcC dopamine were only examined for responses elicited by two solutions. The specific compositions and concentrations of the solutions were selected to build on our prior work demonstrating that VTA sCT reduces intake of both 25% sucrose and 10% Intralipid (Mietlicki-Baase et al., 2017). Because these two solutions are isocaloric, the comparison provided in the current work helps to rule out caloric density as a factor contributing to the differential responses in NAcC dopamine responses revealed here. However, VTA amylin receptor activation suppresses intake of a variety of other foods and fluids as well, including mixed-macronutrient foods like chow and high-fat diet, but also solutions like saccharin, and even stimulated water intake (Mietlicki-Baase et al., 2013, 2015, 2017). Previous work has shown that intra-NAcC administration of a combination of D1 and D2 receptor agonists blunts the ability of VTA sCT to suppress intake of chow or high-fat diet (Mietlicki-Baase et al., 2015). Since pharmacologically activating accumbens dopamine receptors attenuates the hypophagic effect of VTA sCT, this suggests that VTA amylin receptor activation leads to a reduction in dopamine receptor activity in the NAcC to produce hypophagia when a mixed-macronutrient food is available. A similar effect may underlie VTA sCT-induced reductions in the intake of isolated macronutrients as well. However, an important remaining gap in our understanding is whether the effects observed here, or in previous studies evaluating the effects of VTA sCT on intake of isolated macronutrient solutions, also occur for other types of fluid. For example, here we used sucrose as our palatable carbohydrate solution, but it is unknown whether these effects would also occur if glucose was used as the carbohydrate. Similarly, outcomes for other types of fats should be examined in future work. Concentration dependence of these results would also be valuable to investigate. Nevertheless, the present studies provide an important framework and demonstrate differences in NAcC dopamine response to fat and sucrose solutions that should be further explored in future experiments.

In summary these results suggest that baseline differences exist in the DA signals elicited by isocaloric fat and sucrose. Furthermore, amylin receptor activation in the VTA blunts DA release in the NAcC is evoked by random intraoral delivery of both fat and sugar, but DA in response to fat is significantly more attenuated than that to sugar. Taken together, these findings shed new light on the central responses to palatable nutrient solutions and highlight amylin receptors as a therapeutic target for obesity.

## Synthesis

Reviewing Editor: Sam Golden, The University of Washington Decisions are customarily a result of the Reviewing Editor and the peer reviewers coming together and discussing their recommendations until a consensus is reached. When revisions are invited, a fact-based synthesis statement explaining their decision and outlining what is needed to prepare a revision will be listed below. The following reviewer(s) agreed to reveal their identity: Natalie Zlebnik. Note: If this manuscript was transferred from JNeurosci and a decision was made to accept the manuscript without peer review, a brief statement to this effect will instead be what is listed below.

Dear Dr. Mietlicki-Baase,

Your manuscript has been transferred from JN to eNeuro, and the rebuttal letter and revised manuscript reviewed by a original reviewer. Based on their comments and an editorial review, the manuscript has been accepted.
